# The impact of ambient temperature and powertrains of SUVs on the environment in Slovakia during the use phase

**DOI:** 10.1007/s10661-024-12829-2

**Published:** 2024-07-05

**Authors:** Michal Sečkár, Marián Schwarz

**Affiliations:** Department of Environmental Engineering, Faculty of Ecology and Environmental Sciences, Technical University in Zvolen, T. G. Masaryka 24, 960 01 Zvolen, Slovakia

**Keywords:** Life cycle assessment, Energy mix, Ambient temperature, SUV, Emissions

## Abstract

This study compares different powertrains of sport utility vehicles (SUVs) with respect to ambient temperature and energy mix in Slovakia using the well-to-wheel (WTW) Life Cycle Assessment (LCA) method. Battery electric vehicles (BEV), plug-in hybrid electric vehicles (PHEV), and petrol and diesel vehicles were assessed and compared. The WTW study was conducted in SimaPro software assessing electricity/petrol/diesel production, transport, and use (energy conversion in the vehicle), with impact categories being climate change, particulates, NO_x_ emissions, ionizing radiation, and fossil resource scarcity depending on the season (summer and winter). The results indicate that for Slovak conditions, BEV generally had the lowest environmental impact in both seasons studied. The only exceptions were ionizing radiation, which is clearly caused by the high share of nuclear power in the Slovak energy mix, and NO_x_ emissions, which are caused by the combustion of biomass for electricity generation. The other impact categories were dominated by vehicles with an internal combustion engine. The results of emissions from fuel production are also given for each impact category. The transportation of fuel did not exceed the value of 1% for any impact category or for any powertrain. The conclusions of the study support the global trend in favour of vehicle electrification as an important way to reduce the negative environmental impacts of internal combustion engine vehicles in Slovakia.

## Introduction

One of the world’s major problems is emissions from internal combustion engines due to their negative effects on air quality, human health, and global warming. The transport sector accounts for a large part of the world’s CO_2_, CH_4_, and NO_x_ emissions and is therefore one of the largest contributors to global greenhouse gas (GHG) emissions and other environmental impacts. Life Cycle Assessment (LCA) studies are important precisely for understanding different powertrains in terms of vehicle use, as they provide a clear picture of the environmental impacts of a given powertrain over the life cycle associated with the energy process. Well-to-wheel (WTW) studies are a type of LCA study providing comprehensive and relevant information on the environmental impacts of powertrains in terms of rational mobility decision-making. The study deals with the life cycle phases from raw material extraction through production, distribution, and use of the vehicle. This ensures that all relevant environmental impacts associated with propulsion are taken into account. In particular, this study examines the effects of the season and energy mix on different powertrains and their subsequent comparison. Not only vehicles with a combustion engine have a negative impact on the environment but also hybrid (in this study PHEV) and battery electric vehicles (BEV). Although these alternative technologies are considered to be greener, their environmental impact is still significant, especially in the area of fuel and electricity production. The most important phase in GHG emissions is the use and production of fuel/electricity. In this context, it is essential to pay special attention to them. These two areas represent key points where significant improvements in the environmental impacts can be achieved. The use of electric vehicles at low temperatures has a significant impact on the performance and life cycle of lithium-ion batteries used in them. As the temperature decreases, the conductivity of the electrolyte and the charge-transfer kinetics of the battery decrease, leading to an increase in internal resistance and a loss of battery capacity. This has a direct impact on the range, which is significantly reduced as a result of these factors. In addition, the range of electric vehicles will be further reduced due to the increasing energy requirements for the thermal comfort of the electric vehicle cabin at low temperatures (Senol et al., 2023). The impact of low temperatures on emissions from petrol and diesel engines is a serious environmental problem that has significant impacts on air quality and human health. Low ambient temperatures significantly affect emissions of harmful pollutants such as THC (total hydrocarbons), CO, NO_x_, and particulates (Dardiotis et al., 2013). Yusuf and Inambao (2019) studied in their research (including several other scientific studies) how ambient temperature mainly influenced the emissions at car start-ups in motor vehicles. On a cold start, emissions increase several times.

## Materials and methods

**Fig. 1:**
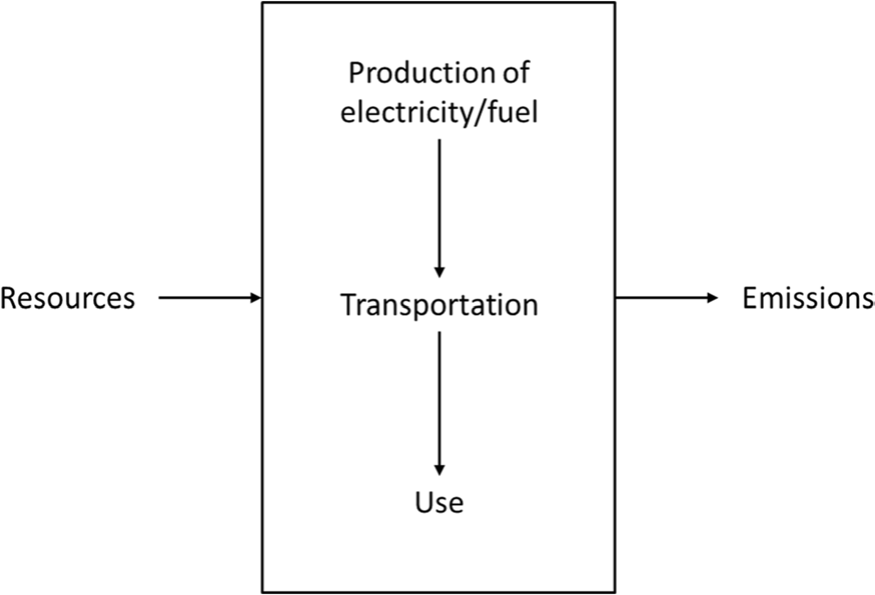
System boundaries

The goal of the study is to compare the environmental impacts of a fully electric, plug-in hybrid vehicle with petrol and diesel vehicles in terms of use in the Slovak Republic. The LCA includes electricity/petrol/diesel production, transport, and use (WTW). The environmental impacts were analyzed within the LCA software SimaPro. Two scenarios were considered - driving 500 km in summer and winter. The current highway network with a total length of 492 km is continuously being expanded in Slovakia (Slovakia Travel, 2024). The distance was chosen because the total distance from the capital Bratislava through Žilina to Košice is 451 km and was rounded to 500 km. The system boundaries have been defined as the actual production, transport, and conversion of fuel/electricity in the vehicle (Fig. 1).

Data on petrol, diesel, electricity, transportation, and use (including indirect emissions from brake, asphalt, and tyre abrasion) were used from the Ecoinvent 3 and USLCI databases, from manufacturers, and from studies by other authors. The impact categories considered are:

- Climate change
- Particulates
- NO_x_ emissions
- Ionizing radiation
- Fossil resource scarcity

For comparison, we selected four powertrain types from one car manufacturer (Table 1). Sport utility vehicles (SUVs) were considered. We selected diesel, petrol, PHEV, and BEV as the powertrains. For the purpose of the study, we selected the CITROËN C5 Aircross for diesel, petrol, and PHEV and the CITROËN - ëC4 X for BEV. All vehicles are approximately at the same performance level, and we consider them as new vehicles. We used the PHEV in this study with a hybrid drive (the electric drive for summer was 56 km and for winter 32 km). We have also relied on emissions data from the technical certificates of new vehicles (Table 2). Emission values for winter are discussed in the following segments.

**Table 1:** Vehicle characteristics

Characteristics	Diesel	Petrol	PHEV (petrol + electricity)	BEV (electricity)
	CITROËN C5 Aircross			CITROËN- ëC4 X
Operating weight (kg)	1 567	1 483	1 845	1 696
Engine displacement (cc)	1 499	1 199	1 598	
Engine power (kW)	96	96	133	
Combined fuel consumption (l/100 km)	5.25	6.15	1.5	
Emission standard	Euro 6	Euro 6	Euro 6	
Electric engine power (kW)			81.2	100
Battery capacity (kWh)			13.2	50
Electricity consumption (Wh/km)			236	139
Battery type			Lithium-ion	Lithium-ion
Declared range (km)			56	360

**Table 2:** Emissions of selected vehicles in use phase (CO_2_, CO, THC, NO_x_, and PM) [g/km] (data used for summer)

Power unit	CO_2_	CO	THC	NO_X_	PM	Source
Diesel	145	0.4440	0.0040	0.0505	0.0002	Manufacturer data (vehicle registration)
Petrol	148	0.4351	0.0244	0.0297	0.0008	Manufacturer data (vehicle registration)
PHEV	32/100*	0.4600	0.0150	0.0160	0.0010	Manufacturer data (vehicle registration)

*Data used in summer according to Plötz et al. (2022)

Fuel/electricity consumption is much higher than stated in the vehicle registration from the manufacturer. In our case, according to Plötz et al. (2022), for PHEV real-world fuel consumption when driving 500 km with 11.2% electric driving share (EDS), we assume a PHEV fuel consumption of 7.5 l/100 km in summer and 10 l/100 km in winter with 6.4% EDS and CO_2_ emissions 100 g/km. According to the authors, in the vehicle registration is stated fuel consumption according to the worldwide harmonized light-duty vehicles test procedure (WLTP), which is 23.3 km with 70-85% EDS. According to the website spritmonitor.de, which collects and provides information on the fuel consumption of vehicles in real conditions, we assume the selected vehicles fuel consumption after driving 500 km for a petrol vehicle of 8.75 l/100 km in the summer and 11.5 l/100 km in the winter. For a diesel vehicle, we assume a fuel consumption value of 5.6 l/100 km in summer and 7.5 l/100 km in winter. For BEV, we assumed an electricity consumption of 20 kWh/100 km in summer and 35 kWh/100 km in winter (Spritmonitor, 2024).

### Petrol and diesel vehicles

The products produced by the ideal combustion of hydrocarbons (HC) in an engine are water (H_2_O), nitrogen oxides (NO_x_), and carbon dioxide (CO_2_), with their mass ratio being a function of the H/C ratio of the fuel. Water is environmentally harmless, and CO_2_ is non-toxic but contributes significantly to the greenhouse effect. Reducing specific fuel consumption reduces CO_2_ emissions during engine combustion. However, combustion in petrol or diesel engines is not ideal and produces additional by-products that are partly harmful to the environment. Imperfect combustion in the homogeneous operation of a combustion engine produces unburned HC but mainly carbon monoxide (CO) as pollutants (Tschoeke et al., 2010).

Several factors influence the efficiency of petrol and diesel vehicles. The most important factors are humidity and ambient temperature. In this study, for vehicles of this type, we consider the effect of outside temperature on the vehicle, the actual production of the fuel, the transport of the fuel from the refinery to the point of use (200 km), and the use of the vehicle over 500 km.

### Ambient temperature and internal combustion engines

Meteorological factors have a significant impact on engine operation, vehicle use, and/or emission control. Ambient temperature influences internal combustion engine emissions. The internal combustion engine at lower temperatures in winter emits more emissions at start-up than in summer conditions. According to Ramadhas and Xu (2016), these facts in diesel engines are caused by higher fuel injection, especially during cold start. Furthermore, they state that the fuel would have higher viscosity and hence higher surface tension at low temperatures. And because of this, the fuel would evaporate slowly, sputter poorly, or could hit the cylinder wall. Also, high heat transfer between the cylinder and the environment could lead to poor combustion. Because of these factors, the fuel would mix poorly with the air, and imperfect combustion would occur, increasing HC, NO_x_, and PM emissions. The authors reported that the warm intake air into the engine improved the fuel combustion and significantly reduced the HC, NO_x_, and PM emission values in cold conditions. Even for petrol vehicles, ambient temperature influences emissions. Ambient temperature has an impact on changes in CO_2_, CO, HC, NO_x_, and PM (not only) emissions (Zhai et al., 2020).

Since cold start and ambient temperature are closely related and have a large influence on each other, we also included cold start emissions in the study. The contribution of cold start for petrol vehicles was also investigated in the work by Pielecha et al. (2021). The authors found that at 8 °C (the lowest temperature studied), the proportion of cold start emissions was highest as a function of total driving emissions. The value of the cold start proportion at 90 km of travel for CO_2_ was 3.4% and for CO 10.7%. For NO_x_, the value of the cold start proportion was 2.9% and for PM 0.1%. At 25 °C, the value of the cold start proportion for CO_2_ was 3.4% and for CO 4.6%. For NO_x_, the value of the cold start share was 3.4% and for PM 0.15%. THC is not reported.

A study by Suarez-Bertoa and Astorga (2018) reports on the impact of cold temperatures on Euro 6 passenger car emissions. Tests were performed using the worldwide harmonized light-duty test cycle (WLTC). The temperatures at which the authors investigated these effects were 23 and −7 °C. They found that petrol cars had on average 6.5 times higher THC emissions in colder conditions. CO emissions were on average 2.6 times higher, NO_x_ emissions were 1.7 times higher, CO_2_ emissions were higher by 9%, and particle number emissions were on average 4.3 times higher in colder conditions. For diesel vehicles, THC emissions are higher on average 1.5 times and for CO emissions 1.8 times in colder conditions. NO_x_ emissions were on average 3.4 times higher, and CO_2_ emissions were higher by 15%. For particle number emissions, they were on average 5 times higher in colder conditions.

In our study, we work with data from the above-mentioned two articles for winter conditions.

### Electric vehicles

The efficiency of electric vehicles also depends on several factors. The biggest factor when buying an electric vehicle is the range, which is limited compared to that of internal combustion engine vehicles. Furthermore, the limited range for EVs decreases in extreme temperatures (Steinstraeter et al., 2021). In this study, we address the impact of ambient temperature on the battery, energy storage efficiency, electricity generation, electricity transport, and vehicle use over 500 km. Powertrains using fossil-based fuels have energy losses that directly translate into vehicle consumption. However, for electric vehicles that are powered when charging from the grid, we account for losses from the distribution network and energy storage efficiency.

### Slovak energy mix

In 2022, the national energy mix in Slovakia was largely made up of nuclear energy sources. Energy generated from nuclear power plants in Slovakia accounts for up to 60.11% of the total energy generated. Hydropower comes second with a share of 14.79%, and energy from natural gas comes third with a share of 8.56%. Energy production from lignite and hard coal accounts for 3.28% and 2.55%, respectively. Petroleum products and other fossil resources account for 3.61% in addition to the above. Biomass energy production accounts for 4.14%, solar energy for 2.57%, and other renewable energy sources for 0.39%. In total, Slovakia produced 24.68 TWh of energy (OKTE, 2023).

### Losses from battery charging

Losses from the grid typically range from 9 to 12%, and lithium-ion batteries have the highest energy storage efficiency (90%) compared to other battery types. The value represents the energy output per energy input to the battery, considering charging and discharging efficiency. Charging rate and energy consumption in the charging phase are jointly related to energy losses during battery charging (Faria et al., 2013; Hawkins et al., 2012). Based on these findings, in this study, we consider a 10.5% energy loss from the grid and an energy storage efficiency of 90%.

### Ambient temperatures and EV/PHEV

One of the most important factors in the range of electric vehicles is cold or hot temperature. The range is reduced due to heating or cooling of the battery and/or the vehicle interior. Moreover, very cold temperatures degrade the battery and impair the ability to store and release energy (Koncar & Bayram, 2021).

Results from Donati et al. (2015) show that energy consumption is dependent on ambient temperature, and the highest consumption values are reached during cold seasons and in cities in cold areas. They compared average energy consumption for four regions (Sweden, Denmark, Ireland and Spain) correlated with average winter and summer ambient temperatures. In hot and cold conditions, consumption increases, due to the use of heating or cooling. According to the study, the optimum ambient temperature for the lowest consumption in terms of heating and cooling ranges from 10 to 20 °C. In addition, the authors found that median energy consumption in the winter months increased by 10% in Spain and up to 75% in Sweden.

Iora and Tribioli (2019) studied the effects of outdoor temperature on the energy consumption and range of a Nissan Leaf electric vehicle. They found that the effects of ambient temperature are remarkable. For the New European Driving Cycle (NEDC), the range at 20 °C was above 150 km. At lower temperatures, it decreased significantly. At 0 °C, the range was 85 km (56.66%), and at −15 °C, it was 60 km (40%).

Al-Wreikat et al. (2022) monitored changes in specific energy consumption (SEC) based on ambient temperature on a Nissan Leaf - BEV. They performed 1 137 runs at temperatures ranging from 0 to 33 °C. They found that the lowest SEC values occur in the months of June and July. Conversely, the highest SEC values occur in December and January. The increase in SEC values from the lowest to the highest average values is up to 69.5%.

We used validated data from the Ecoinvent database for electricity production; no emission factors were used. Only the production of electricity (with losses) needed to travel 500 km was calculated. For the purpose of this study, we have defined electricity consumption based on the studies above. For PHEV and BEV, in winter conditions at 0 °C, we assume a 1.75-fold increase in electricity consumption.

**Fig. 2:**
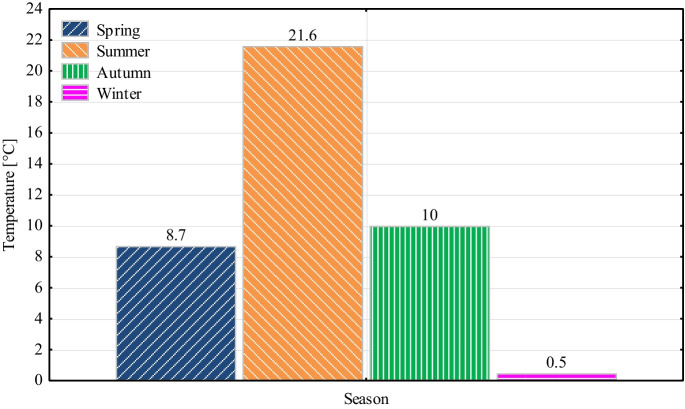
Average air temperature in seasons in 2022 in the Slovak Republic (adapted from SHMÚ, 2023)

As the average summer temperature in Slovakia is 21.6 °C (Fig. 2), the optimum temperature for the use of vehicles, the study deals with comparing the summer and winter seasons.

## Results

The environmental impacts of different vehicle powertrains were compared between summer and winter. The results present the most significant impacts and differences between the powertrains. The study was limited by the production, transport, and fuel/electricity consumption of the vehicle. SimaPro 9.5.0.0 software was used for the analysis, and the ReCiPe 2016 Midpoint (H) V1.08/World (2010) H method was used within the software.

### Global warming

**Fig. 3:**
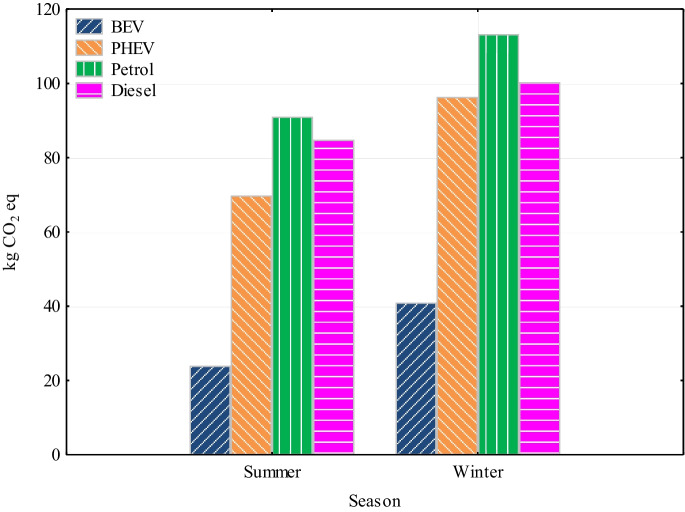
Impacts on global warming

In terms of global warming impacts, the results indicate that BEV and PHEV contribute the least to GHG emissions in both summer and winter (Fig. 3). The lowest value of global warming was 24.1 kg CO_2_ eq in the summer season and 41 kg CO_2_ eq in the winter season for BEV. For BEV, the largest contribution to global warming was for electricity generation from natural gas, lignite, petroleum products, and hard coal (~91%), respectively. PHEVs global warming impact value was 69.8 kg CO_2_ eq in the summer season and 96.4 kg CO_2_ eq in the winter season. The internal combustion engine accounts for the largest share of GHG emissions (winter 68%, summer 73%) in the case of PHEV. The internal combustion engine is switched on when the battery needs to be recharged or when more power is needed. Vehicles with a internal combustion engine were the most significant contributors to greenhouse gas emissions. The highest value for the petrol engine vehicle for the summer season was 91.1 kg CO_2_ eq and 113 kg CO_2_ eq for the winter season. This is associated with the highest fuel consumption of all vehicle types. The diesel vehicle had a value of 84.9 kg CO_2_ eq just behind the petrol vehicle in the summer season and 100 kg CO_2_ eq in the winter season. Global warming emissions associated with petrol vehicle fuel production were 18.72 kg CO_2_ eq (20.5%) in summer and 24.2 kg CO_2_ (21.6%) in winter. The emissions from fuel production of the diesel vehicle were 11.63 kg CO_2_ eq (13.7%) in summer and 15.13 kg CO_2_ (15.1%) in winter. PHEV petrol production emissions were 18.5 kg CO_2_ eq (26.5%) in summer and 30.1 kg CO_2_ eq (31.2%) in winter. Emissions associated with the transportation of fuel were < 1% for these powertrains.

### Particulates

**Fig. 4:**
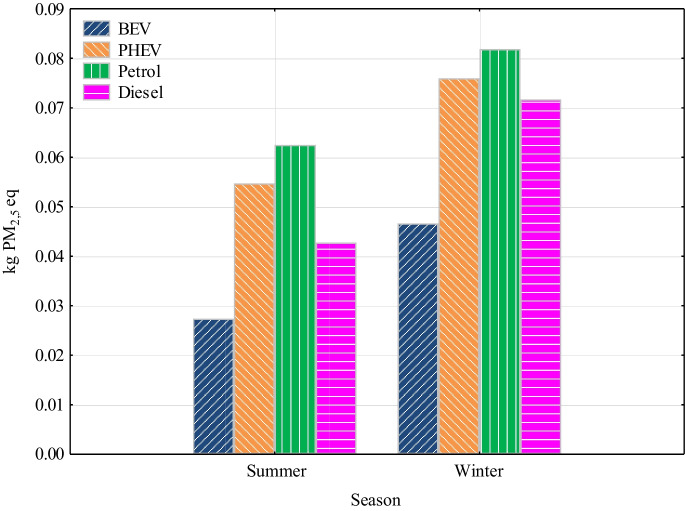
Particulates

Particulate emissions were emitted least from BEV and diesel vehicles in both seasons (Fig. 4). For BEV, in the summer season, the value of particulates was 0.0274 kg PM_2.5_ eq and 0.0467 kg PM_2.5_ eq in the winter season. The diesel vehicle had slightly higher particulate emission values. In the summer season, the diesel vehicle had a particulate value of 0.0428 kg PM_2.5_ eq and in the winter season 0.0717 kg PM_2.5_ eq. In the summer season, the highest particulate matter emission value is at 0.0625 kg PM_2.5_ eq for a vehicle with a petrol engine, and for PHEV, it is at 0.0547 kg PM_2.5_ eq. In winter, the largest particulate matter emitter was the petrol vehicle with a value of 0.0818 kg PM_2.5_ eq. This is due to cold start and greater fuel consumption associated with higher emission rates. The PHEV winter emission value was at 0.0760 kg PM_2.5_ eq. The biggest impact for internal combustion engines and PHEVs in both summer and winter was fuel production. The share of particulate emissions associated with the production of fuel for a petrol vehicle was 96% in summer and 94% in winter. For a vehicle with a diesel engine, it was 92.6% in summer and 70.9% in winter. PHEV’s share of petrol production emissions is 65% in summer and 60% in winter. Emissions associated with the transportation of fuel were < 1% for these power units. For BEV, the largest contribution of particulates was electricity generation from petroleum products and lignite.

### NO_x_

Based on the ReCiPe 2016 Midpoint (H) V1.08/World (2010) H method, NO_x_ emissions are split into two impacts:

- Ozone formation, human health (damage to human health due to ozone)
- Ozone formation, terrestrial ecosystems (terrestrial ecosystem damage due to tropospheric ozone-forming emissions).

**Fig. 5:**
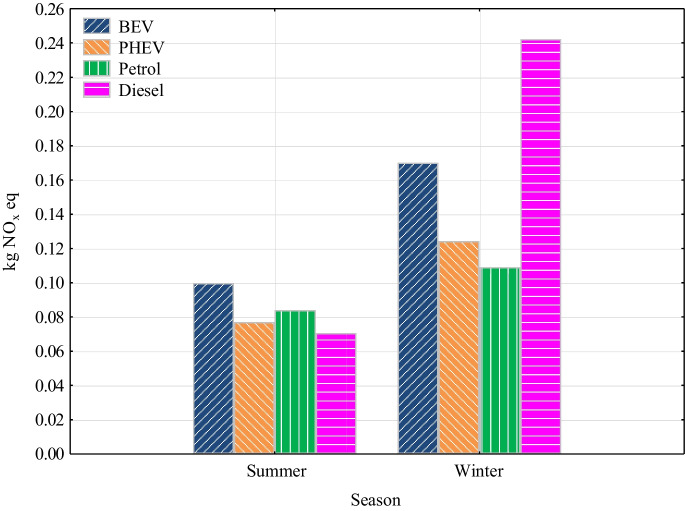
Ozone formation and human health

In terms of human health impacts, we observed the greatest impacts in summer for BEV and in winter for the diesel vehicle (Fig. 5). BEV emissions were 0.1 kg NO_x_ eq in summer and 0.17 kg NO_x_ eq in winter. Diesel vehicle emissions were 0.071 kg NO_x_ eq in summer and 0.242 in winter. PHEV emissions were 0.077 kg NO_x_ eq for summer and 0.124 for winter. The petrol engine vehicle showed relatively the same value as each powertrain in the summer season (0.0841 kg NO_x_ eq), and in the winter season, it was already well behind each powertrain with a value of 0.109 kg NO_x_ eq. The most significant impact for BEV in both seasons was observed for electricity generation from biomass (48.6%), oil (21.6%), natural gas (10%) and other sources (19.8%). For PHEV in both seasons, the most significant impact values were associated with the production of fuel and the use of an internal combustion engine. The use of internal combustion engine in the summer and winter season accounted for 14.4% and 25%, respectively; the share of electricity generation from biomass and oil at together was 12% and 13%, respectively; and the share of fuel production and other processes was 73.6% and 62%, respectively. For internal combustion engine-only vehicles, the largest impact is associated with vehicle use and fuel production. The share of vehicle use in summer and winter for a petrol engine vehicle was 21.4% and 20.6%, respectively; the share of fuel production was 77.6% and 78.7%, respectively; and the share of fuel transport was only 1% and 0.7%, respectively. In case of diesel vehicle, in the summer season, 36.5% of the impact was attributed to vehicle use and 62.5% to fuel production and other processes. In contrast, in winter, up to 75.2% of the total impact was associated with vehicle use, with only 23.8% attributed to fuel production. Emissions associated with transportation of fuel were 1%.

**Fig. 6:**
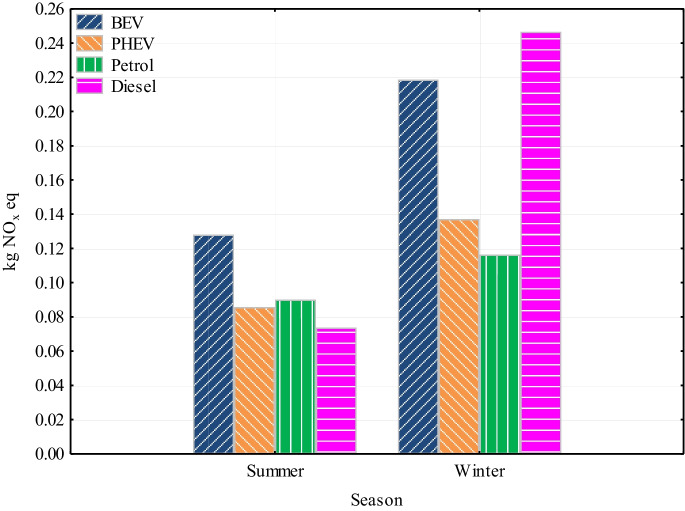
Ozone formation and terrestrial ecosystems

The values for NO_x_ impacts on terrestrial ecosystems were approximately the same as for human health impacts (Fig. 6). In the summer season, the BEV showed the highest impact value (0.128 kg NO_x_ eq), followed by the petrol engine vehicle (0.0902 kg NO_x_ eq), the PHEV (0.0858 kg NO_x_ eq), and the diesel vehicle (0.0739 kg NO_x_ eq). In the winter season, the highest value was for the diesel vehicle (0.246 kg NO_x_ eq). This is mainly related to the cold start of the diesel engine, which has a large impact on NO_x_ emissions. Followed by BEV (0.219 kg NO_x_ eq), PHEV (0.137 kg NO_x_ eq), and finally petrol engine vehicle with the lowest impact (0.116 kg NO_x_ eq). The highest impacts for BEV in both seasons were electricity generation from biomass (59.4%), oil (17%), natural gas (8.2%), and other sources (15.4%). For PHEV, in both observed seasons, the biggest impacts were attributed to the use of internal combustion engine and fuel production. The percentage ratio of the use of combustion engines was in summer and winter, respectively, 15% and 24%; the production of electricity from biomass and oil was 15.1% and 15.5%, and the production of petrol and other processes was 68.9% and 59.5%. For both internal combustion engine-only vehicles, the biggest impact was associated with car use and fuel production. The share of use for petrol engine vehicles in summer and winter was 22% and 21%, fuel production was 77% and 78.3%, and fuel transport was only 1% and 0.7%, respectively. For the diesel engine vehicle, the ratio of values between fuel use and fuel production has changed compared to the petrol engine vehicle. In the summer season, 35.5% of the impact was attributed to vehicle use and 63.5% to fuel production and other processes. However, in the winter season, up to 74.4% was attributed to car use and only 24.6% to fuel production. As mentioned before, this is linked to the cold start of the vehicle in the winter. Emissions associated with transportation of fuel were 1%.

### Ionizing radiation

**Fig. 7:**
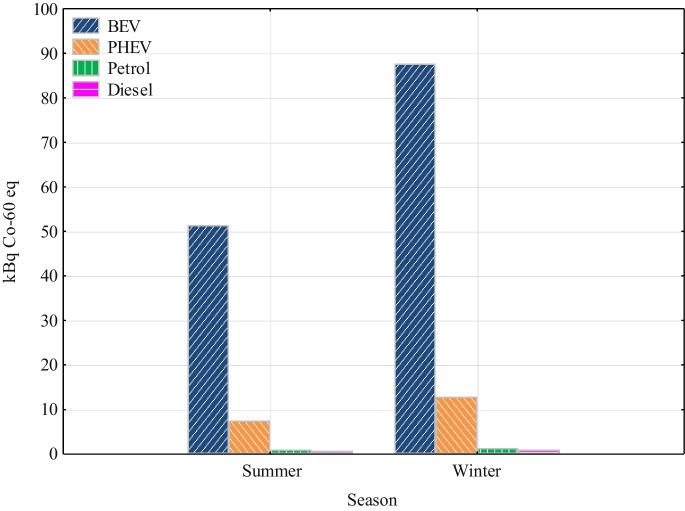
Ionizing radiation

The ionizing radiation results show the only impact category examined where BEV have the greatest environmental impact in both seasons (Fig. 7). The ionizing radiation (activity) value for BEV was 51.4 kBq Co-60 eq in the summer and 87.6 kBq Co-60 eq in the winter. For PHEV these are lower values. PHEV had a value of 7.55 kBq Co-60 eq in the summer season and 12.9 kBq Co-60 eq in the winter season. For diesel and petrol vehicles in both winter and summer, the value was around 1 kBq Co-60 eq. The BEV and PHEV values are higher due to the energy mix of Slovakia, where up to 60.11% of the electricity is generated from nuclear power. Despite the seemingly excessive radiation burden due to the high proportion of electricity generated from nuclear power, the dose equivalent of ionizing radiation to the population is < 0.01 mSv for living near a nuclear power plant compared to other radiation sources according to the US EPA (EPA, 2015).

### Fossil resource scarcity

**Fig. 8:**
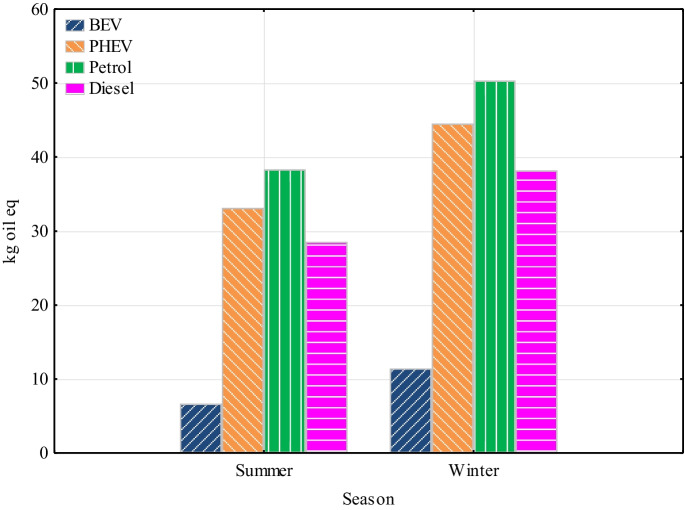
Fossil resource scarcity

The fossil fuel potential (higher heating value) was used as midpoint indicator. Within this impact category, cars with internal combustion engines had the largest effect in both seasons (Fig. 8). In the summer season, petrol and diesel engine vehicles had impact values of 39.3 and 28.5 kg oil eq, respectively. In the winter season, the impact values were for petrol vehicle 50.4 kg oil eq and for diesel vehicle 38.2 kg oil eq. The impact values for the PHEV were 33.1 and 44.5 kg oil eq for the summer and winter seasons, respectively. For BEV, the value for the summer season was 4.66 and for the winter season 8.15 kg oil eq. The highest contribution for all powertrains is due to fuel production in both seasons. For BEV, 51.5% of the impact was electricity generation from natural gas alone in both seasons. As PHEV use both an electric and an internal combustion engine, the results have to be further specified. For the summer season, the share of the internal combustion engine was up to 93% and for the winter season up to 92%. The results show that internal combustion engines had a significantly greater impact than electric vehicles in terms of fossil resource scarcity. The impact associated with transportation of fuel was < 1%.

## Discussion

According to Egede et al. (2015), there are very few studies that address the use phase. Most of the studies deal with vehicle production and related raw material extraction. Furthermore, the authors also mention the conditions that affect the environmental impacts of electric vehicles (EVs) during their use. Climatic conditions (temperature, air speed and direction, precipitation, humidity, etc.) affect the need for heating and cooling interior and there are significant variations in energy consumption at different times of the year. A thorough environmental impact assessment using LCA helps to identify cases and areas where EVs have specific benefits that compete with internal combustion engine vehicles.

A very important factor in assessing environmental impacts and comparing BEVs and PHEVs with internal combustion engine-only vehicles is the energy mix of the country. A study by Rangaraju et al. (2015) reports the impacts of BEVs according to the Belgian energy mix. The results show that BEVs have lower emissions of CO_2_ eq, SO_2_, NO_x_, and particulates than petrol and diesel vehicles, which is due to the large share of nuclear energy in Belgium energy mix, similar to the case of Slovakia. The authors used annual average emissions to assess the energy mix whereas in our case the energy mix was modelled with SimaPro software, and the two results for WTW correlate with each other. By evaluating the global warming impacts, it was found that petrol engine vehicles showed the highest values, while diesel engine vehicles showed the highest NO_x_ values. In the case of particulates, we found in our study that higher values are reached in both seasons for petrol engines (as in the Belgian study) (the authors did not make such a comparison in winter).

Petrauskienė et al. (2020) investigated the impact of energy mix in Lithuania during 2015-2050 (changing every 5 years) on environmental components using a Cradle-to-Grave LCA method followed by a comparison with conventional vehicles. The energy mix consisted of electricity generation from natural gas (41.73%), hydropower (20.55%), wind power (14.56%), biomass (5.85%), geothermal (5.19%), oil (4.57%), biogas (3.51%), waste (2.28%), and solar (1.76%). In terms of GHG emissions for the use phase (WTW + maintenance), the authors report the largest impact for BEVs, which is due to the high share of electricity generation from natural gas. When assessing the impact of ionizing radiation, large differences were found due to the absence of electricity generation from nuclear power, and at the same time, BEV values for the use phase were several times lower compared to vehicles with internal combustion engines. This is quite the opposite phenomenon to the Slovak case. The results of the authors in the assessment of fossil depletion correspond with our results.

In the study by Lieutenant et al. (2022), the authors address an LCA case study of GHG emissions with respect to different powertrains in some European countries (Poland, Norway, Germany, and Romania), which are compared with the European average. Poland shows the worst of all scenarios, where up to 83% of electricity is generated from fossil fuels (71.2% coal, 2.9% oil, and 9.3% natural gas), and the best option here appears to be a petrol engine vehicle, which had 22.5% lower GHG emissions when driving 200,000 km than BEV (diesel was not compared).

## Conclusion

The study was undertaken by assessing the environmental impacts of different powertrains over two seasons using the LCA software SimaPro. The study is limited only to the use phase and production and transport of energy and fuels. The conclusions of the study indicate that the choice of powertrain of vehicles significantly affects their environmental impact, especially in terms of impacts on global warming, particulates, NO_x_ emissions, ionizing radiation and fossil fuel scarcity. In the study, vehicles with an electric drive stand out the best for Slovakia. The results are assessed as follows:

- The study analyzed a spectrum of impact categories and presented the results of the potential environmental impacts of passenger cars on the components of the environment, thus providing a tool for decision-making on the environmental and health impacts of vehicles in Slovakia.
- In both seasons, BEV was significantly more environmentally friendly compared to vehicles with internal combustion engine.
- BEV and PHEV had the lowest global warming impact, with the most significant difference in GHG emissions coming from vehicle use.
- In the area of particulates and NO_x_, BEV had the lowest values again. Internal combustion engine vehicles, especially in winter, reaching significantly higher values. This trend was most pronounced for diesel vehicles, which showed several times higher values for NO_x_ compared to other types of powertrains. The largest impact for BEV in both seasons was electricity generation from biomass (59.4%).
- In the field of ionizing radiation, SimaPro showed a several times higher environmental impact of BEV and PHEV due to the Slovak energy mix with a high share of nuclear energy.
- Regarding the scarcity of fossil fuels, the results indicate that internal combustion engine vehicles had a significantly higher environmental impact in terms of consumption of these resources compared to electric vehicles in both seasons considered.
- Last but not least, the PHEV showed high values of impact categories for the determined distance. They were almost the same, and in some cases even higher, than with internal combustion engine-only vehicles, with the exception of the global warming impact category.
- Overall, the results of the study support the trend towards vehicle electrification as a way to reduce the negative environmental impact of vehicles in Slovakia. Electric drives appear to be an effective solution for reducing GHG emissions, particulates, and NO_x_ and minimizing the impact of fossil resource scarcity.

### Employment

The authors declare no employment by an organization that may gain or lose financially.

## Data Availability

The data supporting the findings of this study are available within the article.
